# Transient Receptor Potential C 1/4/5 Is a Determinant of MTI-101 Induced Calcium Influx and Cell Death in Multiple Myeloma

**DOI:** 10.3390/cells10061490

**Published:** 2021-06-13

**Authors:** Osama M. Elzamzamy, Brandon E. Johnson, Wei-Chih Chen, Gangqing Hu, Reinhold Penner, Lori A. Hazlehurst

**Affiliations:** 1 Clinical and Translational Sciences Institute, School of Medicine, West Virginia University, Morgantown, WV 26506, USA; omelzamzamy@mix.wvu.edu; 2 WVU Cancer Institute, West Virginia University, Morganton, WV 26506, USA; weichih.chen@hsc.wvu.edu (W.-C.C.); michael.hu@hsc.wvu.edu (G.H.); 3 Center for Biomedical Research, The Queen’s Medical Center, Honolulu, HI 96813, USA; brajohnson@queens.org (B.E.J.); rpenner@hawaii.edu (R.P.); 4 Department of Pharmaceutical Sciences, School of Pharmacy, West Virginia University, Morganton, WV 26506, USA; 5 Department of Microbiology, Immunology and Cell Biology, School of Medicine, West Virginia University, Morgantown, WV 26506, USA; 6 Department of Cell and Molecular Biology, University of Hawaii, Honolulu, HI 96813, USA

**Keywords:** multiple myeloma, SOCE, calcium, TRPC

## Abstract

Multiple myeloma (MM) is a currently incurable hematologic cancer. Patients that initially respond to therapeutic intervention eventually relapse with drug resistant disease. Thus, novel treatment strategies are critically needed to improve patient outcomes. Our group has developed a novel cyclic peptide referred to as MTI-101 for the treatment of MM. We previously reported that acquired resistance to HYD-1, the linear form of MTI-101, correlated with the repression of genes involved in store operated Ca^2+^ entry (SOCE): PLCβ, SERCA, ITPR3, and TRPC1 expression. In this study, we sought to determine the role of TRPC1 heteromers in mediating MTI-101 induced cationic flux. Our data indicate that, consistent with the activation of TRPC heteromers, MTI-101 treatment induced Ca^2+^ and Na^+^ influx. However, replacing extracellular Na^+^ with NMDG did not reduce MTI-101-induced cell death. In contrast, decreasing extracellular Ca^2+^ reduced both MTI-101-induced Ca^2+^ influx as well as cell death. The causative role of TRPC heteromers was established by suppressing STIM1, TRPC1, TRPC4, or TRPC5 function both pharmacologically and by siRNA, resulting in a reduction in MTI-101-induced Ca^2+^ influx. Mechanistically, MTI-101 treatment induces trafficking of TRPC1 to the membrane and co-immunoprecipitation studies indicate that MTI-101 treatment induces a TRPC1-STIM1 complex. Moreover, treatment with calpeptin inhibited MTI-101-induced Ca^2+^ influx and cell death, indicating a role of calpain in the mechanism of MTI-101-induced cytotoxicity. Finally, components of the SOCE pathway were found to be poor prognostic indicators among MM patients, suggesting that this pathway is attractive for the treatment of MM.

## 1. Introduction

Multiple myeloma (MM) is a malignancy arising from the neoplastic proliferation of plasma cells in the bone marrow. MM is the second-most common form of blood cancer accounting for 17% of hematological malignancies, with an estimated 34,920 cases in the United States in 2021, and 12,410 deaths [1,2,3]. Multiple new therapies emerged recently for the treatment of MM, yet unfortunately MM patients continue to progress toward a refractory disease and thus MM remains incurable with current treatment strategies and novel treatment strategies are required to improve patient outcomes.

Cytosolic Ca^2+^ homeostasis is essential for multiple physiological functions, including metabolism, protein phosphorylation, cell proliferation, gene transcription, homing and contractility, and thus the coordinated regulation of Ca^2+^ channels, pumps, transporters, and exchangers is essential for survival [4,5]. Cytoplasmic intracellular Ca^2+^ concentration ranges between 10 and 100 nM, while the extracellular Ca^2+^ concentration is 1.2 mM [6]. Multiple Ca^2+^ channels, pumps, transporters, and exchangers are located on the plasma membrane and on cytoplasmic organelles membranes and function to maintain the Ca^2+^ gradient across the plasma membrane and equilibrate Ca^2+^ intracellularly [5]. Ca^2+^ depletion of intracellular stores triggers the influx of extracellular Ca^2+^ through Ca^2+^-permeable channels in the plasma membrane, a mechanism termed store-operated Ca^2+^-entry (SOCE). This pathway is considered the major Ca^2+^ entry pathway in many non-excitable cells. 

The core of the SOCE pathway is the Ca^2+^ release-activated Ca^2+^ (CRAC) channels located in the plasma membrane. These CRAC channels can form homo- or heteromeric channels encoded by three genes Orai1-3, of which Orai1 is most predominantly expressed. When ER Ca^2+^ stores become depleted, ER-bound Ca^2+^-sensing partners stromal interaction molecules (STIM1 and STIM2) multimerize and translocate to the ER-PM junction. STIM1 interacts with Orai subunits, forming a Ca^2+^-conducting channel complex that functions to elevate cytoplasmic Ca^2+^ and replenish ER Ca^2+^ stores [7,8]. In some cells, Ca^2+^ entry can be modulated by TRPC channels that also interact with STIM [9]. The SOCE pathway functions to increase cytoplasmic Ca^2+^ following signal transduction of several pathways: (i) agonist-targeted G protein-coupled receptors (GPCR); (ii) ligand-target receptor tyrosine-kinases (RTK), and (iii) engagement of lymphocyte antigen receptors. Physiological activation of these receptors leads to the activation of PLC and subsequent hydrolysis of phosphatidylinositol 4,5-bisphosphate (PIP2), generating inositol 1,4,5-trisphosphate (IP_3_), which in turn directly activates the IP_3_ receptor (IP3R), mediating Ca^2+^ release from the ER lumen and active store depletion [10]. The sarcoplasmic/endoplasmic reticulum Ca^2+^ ATPase (SERCA) maintains low cytoplasmic Ca^2+^ levels by pumping Ca^2+^ into the SR and ER lumens. Pharmacological inhibition of the SERCA by thapsigargin (Tg) or cyclopiazonic acid (CPA) prevents ER refilling and enables leak pathways to passively deplete stored Ca^2+^ leading to the activation of the SOCE pathway [11]. 

STIM1/Orai1 co-localization generates inwardly rectifying and highly selective Ca^2+^ currents known as CRAC currents (I_CRAC_) [12].I_CRAC_ can be triggered by depleting ER Ca^2+^ stores with SERCA pump inhibitors (Tg, and CPA), chelating cytoplasmic Ca^2+^, or by applying IP_3_ to the internal side of a cell during a patch-clamp recording [13,14,15]. On the other hand, STIM1/TRPC co-localization may generate a non-selective, cationic channel that allows entry of Ca^2+^ and Na^+^, and has been referred to as the I_SOC_ current [12,16]. Activation of GPCR or RTKs can signal laterally in the membrane and augment signals in a context specific manner [17]. For example, integrin signaling has been shown to augment both growth factor receptor signaling and GPCR signaling. However, integrin signaling also changes multiple downstream pathways, including MAPK, AKT, FAK, and the formation of focal adhesion, leading to cytoskeletal rearrangements [17]. Thus, ligand stimulated activation of I_CRAC_ in the context of interactions with the tumor microenvironment is likely more complex when compared to isolation of the I_CRAC_ signaling cascade, i.e., via specific pharmacological tools, including cell permeable IP_3_ and blocking of the SERCA pump [18,19,20]. TRPC1 can form heteromeric channel assemblies with TRPC4 and TRPC5 subunits, contributing to Ca^2+^ entry, and the activation of these channels remains poorly understood [21,22,23].

We previously reviewed the role of TRPC1 as a function of tumor progression and the hallmarks of cancer [24]. Multiple studies have reported the role of TRPC1 in cancer proliferation, migration, and invasion in pancreatic, lung, breast, colorectal, and multiple myeloma cancers [25,26,27,28]. However, the mechanism by which TRPC channels and the SOCE pathway contribute to these pathophysiological outcomes is not fully understood. Furthermore, there is a pharmacological opportunity to consider this pathway as a strategy for novel cancer treatment. [21]. HYD-1 is a D-amino acid linear peptide that was originally discovered to inhibit tumor cell adhesion to immobilized extracellular matrix proteins [29]. We later reported that HYD-1 and the more potent cyclic peptide MTI-101 induced necrotic cell death through depolarization of the mitochondrial membrane potential, reactive oxygen species (ROS) production, and ATP depletion[30,31]. To further understand the mechanism and determinants of resistance, we developed a HYD-1-acquired drug resistant MM cell line. A comparative gene expression profiling between the parental drug sensitive and resistant variant revealed changes in genes encoding for multiple components of Ca^2+^ entry pathways, including TRPC1, TRPM7, ATP2A3, PLCB, and ITPR3 [32]. The cell line was selected for resistance to the linear peptide HYD-1, but the resistant variant was cross-resistant to MTI-101 induced cell death. Finally, we showed that HYD-1 and MTI-101 induced increased Ca^2+^ influx, and that relapsed multiple myeloma patients’ specimens were more sensitive to treatment with MTI-101 when compared to specimens obtained from newly diagnosed patients [32]. The non-specific SOCE inhibitor 2-APB attenuated MTI-101-induced cell death in MM cell lines and in patients’ specimens. Translationally, MTI-101 synergized with MM standard of care treatment bortezomib in C57BL/KaLwRijHsd mice, where the combination treatment significantly increased survival [32]. In this study, we sought to further characterize the role of STIM1-TRPC heteromers in contributing to MTI-101 induced cell death. 

## 2. Materials and Methods

### 2.1. Cells and Reagents

U266 (ATCC Cat# TIB-196) and MM.1s (ATCC Cat# CRL-2974) multiple myeloma (MM) cells, and HS-5 (ATCC Cat# CRL-11882) bone marrow stroma cells were purchased from American Type Culture Collection (Manassas, VA, USA), they were maintained in RPMI-1640 medium with L-glutamine (Gibco; Life Technologies, Waltham, MA, USA) with 10% Fetal Bovine Serum (FBS) and 1% penicillin/streptomycin. The cell lines were validated by short tandem repeat (STR) and they were also tested for mycoplasma every six months. MTI-101 was synthesized by Bachem (San Diego, CA, USA). Thapsigargin (Tg) (Millipore Sigma, Burlington, MA, USA) was used to inhibit the SERCA pumps, calpeptin (Selleckchem, Houston, TX, USA) was used to inhibit calpain I/II, SKF96365 (Selleckchem, Houston, TX, USA) or ML204 (Selleckchem, Houston, TX, USA) were used to inhibit TRPC channels, and GSK7975A (Millipore Sigma Burlington, MA, USA) was used to inhibit CRAC channels.

### 2.2. Ca^2+^ Imaging and Cell Death Assay

Intracellular Ca^2+^ levels were measured in U266, MM.1s, and HS-5 cell lines using the Fluo-4-AM calcium indicator dye. Briefly, cells were seeded at 7.5 × 10^5^ cells per ml in their respective media with 2.5 µM Ca^2+^ sensitive dye Fluo-4-AM (Life Technologies, Waltham, MA, USA) for 45 min in 37 °C in 5% tissue culture incubator, cells were washed and resuspended in media to allow to de-esterification for 30 min. Cells were then resuspended in either Live Cell Imaging Solution (molecular probes; Life Technologies, Waltham, MA, USA), 50 mM KCl physiological saline solution (PSS) or 5 µM CaCl_2_ PSS, N-methyl-d-glucamine (NMDG)-Ringer, or Na^+^ Ringer solutions. All solutions contained 1 µg/mL DAPI (Fisher Scientific, Waltham, MA, USA) as an indicator of cell death. The 50 mM PSS contained (in mM): NaCl 93, KCl 50, CaCl_2_ 2.5, MgCl_2_ 1.2, glucose 7.7, HEPES 10, and PH at 7.2 by NaOH, while 5 µM CaCl_2_ PSS contained (in mM): NaCl 140, KCl 3, and CaCl_2_ at 5 µM, NMDG- Ringer contained (in mM): NMDG 140, KCl 3, CaCl2 2,5, MgCl_2_ 1.2, glucose 7.7, HEPES 10, and PH at 7.2 by NaOH, Na^+^ Ringer contained (in mM): NaCl 140, KCl 3, CaCl2 2,5, MgCl_2_ 1.2, glucose 7.7, HEPES 10, and PH at 7.2 by NaOH. Cells were plated in black-side, clear bottom 384-well plates (Nunc, Fisher Scientific, Waltham, MA, USA), pre-coated with Cell-Tak (Corning, Fisher Scientific, Waltham, MA, USA) at 3 µg/cm^2^ in NaHCO_3_ per manufacturer’s instructions. Ca^2+^ fluorescence by Fluo-4-AM was captured using excitation and emission of 488, and 510, respectively. Cell death was measured by DAPI using excitation and emission of 377, 447 respectively. Images were captured using the BIOTEK Cytation 5 imager. Ca^2+^ imaging was performed every 30 s for 60 min. Captured images were analyzed using Nikon NIS Elements AR software. Images of individual cells (*n* = 50) were traced over time and analyzed for their Fluo-4-AM and DAPI fluorescence. Data presented in graphs show single cell Fluo-4-AM signal as a representative for 50 cells, while the mean of 50 cells was used to determine the peak area under the curve (AUC), and the maximum peak intensity. To correlate the level of intracellular Ca^2+^ with induction of death, experiments were done as mentioned above, but with imaging every 5 min under the same conditions. Data analysis was performed using the BioTek GEN 5.0.3 software, where the threshold of DAPI fluorescence was set to capture only dead cells, and all data in the field of the image were analyzed and normalized to the total cell count in the same field of view. 

### 2.3. DiBAC_4_(3) Membrane Potential Measurement 

Cells were plated in a 384-well plate pre-treated with Cell-Tak (Corning, Fisher Scientific, Waltham, MA, USA) at 25,000 cells/well. Cells were then loaded with 250 nM DiBAC4(3) (Bis-(1,3-Dibutylbarbituric Acid) Trimethine Oxonol) (Thermo Fisher, Waltham, MA, USA) for 20 min in 37 °C in 5% tissue culture incubator. DiBAC4(3) was added to cells in Live Cell Imaging Solution and 50 mM KCl physiological saline solution (PSS) (as mentioned above). The indicator was removed, and cells were loaded with their respective buffer. Membrane potential fluorescence indicated by DiBAC4(3) was captured using excitation and emission of 488 and 510, respectively, using the BIOTEK Cytation 5 imager. Fluorescence was captured every 30 s and analyzed as mentioned previously. 

### 2.4. Quantitative Real-Time PCR (qRT-PCR)

qRT-PCR was performed as previously described [32]. Briefly, 5 µg of total RNA was reverse transcribed using SuperScript III First-Strand Synthesis (Thermo Fisher, Waltham, MA, USA) following Total RNA extraction by Trizol (Invitrogen, Thermo Fisher, Waltham, MA, USA). Real-time PCR was performed on the ABI Prism 7500 Fast instrument (Applied Biosystems, Thermo Fisher, Waltham, MA, USA) using Sybr Green I Mastermix (Thermo Fisher, Waltham, MA, USA). Samples were tested in triplicates, three independent times and GAPDH was used as endogenous control product. Primer sequences were as follows: GAPDH forward 5′-GCATCTTCTTTTGCGTCGCC-3′ and reverse 5′-GCGCCCAATACGACCAAATC-3′, TRPC1 forward 5′-GAGCAGAGGATGACGTGAGG-3′ and reverse 5′-CCCAGGAAGAGGACGAGAGA-3′, TRPC4 forward 5′-AACAATAGGGAGGCGAGCTG-3′ and reverse 5′-CGGTCAGGOCCTTCTTCAGTT-3′, and TRPC5 forward 5′-GCCACACCTTGIAGGACCTC-3′ and reverse 5′-CTGCCCACGTACACTAAGCA-3′. 

### 2.5. Small Interfering RNA Transfection

The transfection of U266 and MM.1s cell lines were done using the Lonza 4D-Nucleofector system (Lonza, Basel, Switzerland). Briefly, 2 × 10^6^ cells were resuspended in the Lonza SF-kit (Lonza, V4XC) with 10 µl of 20 µM stock of the respective small interfering RNA (siRNA). siRNA concentration was determined as previously reported [31]. Cells along with siTRPC4 (Dharmacon, Horizon Discovery, Waterbeach, United Kingdom, ON-TARGETplus SMARTpool), siTRPC5 (Santa Cruz Biotechnology, Dallas, TX, USA, sc-42670), siTRPC1 (Santa Cruz Biotechnology, Dallas, TX, USA, sc-42664), and siControl (Dharmacon, Horizon Discovery, Waterbeach, United Kingdom, ON-TARGETplus Non-targeting Pool) were added along with buffer to the cuvette and electroporated at the recommended settings by LONZA (Basel, Switzerland). Cells were incubated in the buffer for 10 min at 37 ° C, cells were then transferred to a T25 flask in 5 mL of media and incubated for 96 h.

### 2.6. Fura-2AM Based Ca^2+^ Imaging

Cells were loaded with 4.0 μM Fura-2-AM, then plated in a 96-well plate at a density of 60,000 cells/well and fluorescence measurements were made on a Hamamatsu FDSS7000EX kinetic plate reader. Fura-2 was excited at 340 and 380 nm, and the ratio of the respective emission fluorescence at 510 nm was determined. Sampling interval was 3 s. The change in fluorescence ratios from baseline were reported. Bath solutions contained (in mm): NaCl 140, KCl 5.4, CaCl_2_ 2, MgCl_2_ 0.8, glucose 10, and HEPES 20 (pH 7.2 with NaOH). Stock solutions of thapsigargin (Tg) were prepared in DMSO at a concentration of 1 mM. 

### 2.7. Membrane Proteins Biotinylation

The isolation of membrane proteins in U266 cells was done using the Pierce™ Cell Surface Biotinylation and Isolation Kit (Thermo Fisher, A44390). In brief, 1 × 10^6^ cells/mL were treated with MTI-101 (20 µM) for 10 min in Live Cell Imaging Solution followed by resuspension in the Biotin containing solution. Cells were then lysed with the kit’s lysis buffer. A BCA assay was done to ensure equal loading of protein (1mg) to the NeutrAvidin Agarose beads. The elute from the beads and control whole cell lysates were subjected to Western blot analysis. 

### 2.8. Co-Immunoprecipitation

Co-immunoprecipitation protocol was adapted from Hofmann et al. [33]. Briefly, 10 × 10^6^ cells were treated with 20 µM of MTI-101 for 10 min in Live Cell Imaging Solution (Molecular Probes by Life Technologies), centrifuged at 4 °C and lysed with 1% Triton X-100 (Thermo Scientific; 85111) in PBS, with protease and phosphatase inhibitors cocktail (Millipore Sigma). BCA assay was used to quantify protein concentration. 1 mg of protein for each treatment and control groups were incubated with the primary antibody (TRPC1; Santa Cruz Biotechnology, and host species IgG) overnight rotating in 4 °C. Later, protein A/G plus agarose (Santa Cruz Biotechnology; sc-2003) was added to the sample and allowed to incubate rotating in 4 °C for one hour. Samples were washed 3 times in lysis buffer and samples were subjected to Western blot analysis.

### 2.9. TRPC1 Cleavage Analysis

U266, MM.1s, or HS-5 cells were seeded at a density of 7 × 10^5^ cell/mL in 10 mL Live Cell Imaging solution at 37 ºC in 5% tissue culture incubator. 15 µM MTI-101, 2 µM thapsigargin, and 80 µM calpeptin were used to treat appropriate samples and cells were collected at 5, 30, 60, and 90 min post-treatment. Following drug treatment, cells were lysed using 1× RIPA buffer (Thermo Fisher) with protease and phosphatase inhibitors cocktail (Protease Inhibitor Cocktail Set III, Millipore Sigma; 539135, and Phosphatase Inhibitor Cocktail Set II, Millipore Sigma; 524625) for 30 min on ice. Lysates were spun down and supernatant was taken off, the pellet was then resuspended in 1× RIPA buffer plus 2% SDS and lysed for 30 min on ice. Pellets was then sonicated using a probe homogenizer. Protein concentration from both compartments were measured using Pierce BCA Protein analysis kit, and 30 µg were analyzed using Western blot. 

Cleavage of TRPC1 using recombinant calpain-1 was adopted from Kaczmarek et al. In short, U266 and HS-5 cells were collected at 7 × 10^5^ cells/mL, resuspended and sonicated in Live Cell Imaging Solution (Molecular Probes; Life Technologies). Lysates were incubated in the presence of 2.5 mM CaCl_2,_ 50 ug/mL recombinant calpain-1 (LSBio) and in the presence or absence of 80 µM calpeptin. Extracts were incubated at room temperature for 1 h to allow for enzymatic cleavage. After the incubation, cell lysates were subjected to Western blot analysis for the detection of full length and cleaved TRPC1. 

### 2.10. Survival Analysis with Multiple Myeloma Patients

Overall survival analysis was done using expression data (in transcripts per million) and overall survival records (in days) from newly diagnosed MM patients enrolled in the CoMMpass trial (release IA14), downloaded from the Multiple Myeloma Research Foundation researcher gateway (https://research.themmrf.org/, accessed on 19 April 2021). Additional overall survival analysis was conducted using microarray expression data and overall survival records (in days) for patients with relapsed myeloma obtained the APEX/SUMMIT trail (GEO accession #: GSE9782) [34]. The survival curves were generated with the survival R package, and P value was calculated by log-rank test.

### 2.11. Statistical Analysis

Cell death percentage is reported as a representative of experiment performed in quadruplicate, one-way ANOVA was used to determine significance (*p* < 0.05), and Tukey’s multiple comparisons test was used to determine significance between groups. Ca^2+^ and Na^+^ influx measurements were done by measuring the fluorescence intensity of 50 cells and subtracting the baseline fluorescence intensity. We reported the mean of 50 cells with error bars representing the standard error of the mean (SEM), and a single cell that fell in the median. Response magnitudes were quantified by measuring the Maximum Peak and integrating the area under the curve (Peak AUC), respectively, for the first 30 min. Peak AUC was measured using GraphPad Prism (GraphPad Prism, RRID:SCR_002798) by following the trapezoid rule using the equation ΔX*([(Y1 + Y2)/2]−Baseline] to calculate the area. Significance was determined by one-way ANOVA (*p* < 0.05), and Tukey’s multiple comparisons test was used to determine in-between groups significance. All experiments were done three independent times, and shown is a representative experiment. mRNA expression analysis was performed by quantitative real-time PCR 3 independent times from 3 different days in triplicates. We reported the mean of the three experiments by calculating delta CT (Cycle Threshold) and subtracting the CT of the mRNA of interest from GAPDH. For siRNA knockdown experiments, we reported the fold change in expression by measuring the ratio of siRNA of interest to the siControl. We reported the mean of three independent experiments as well. 

## 3. Results

### 3.1. MTI-101 Induces Sustained Ca^2+^ Influx Leading to Cell Death

**Figure 1:**
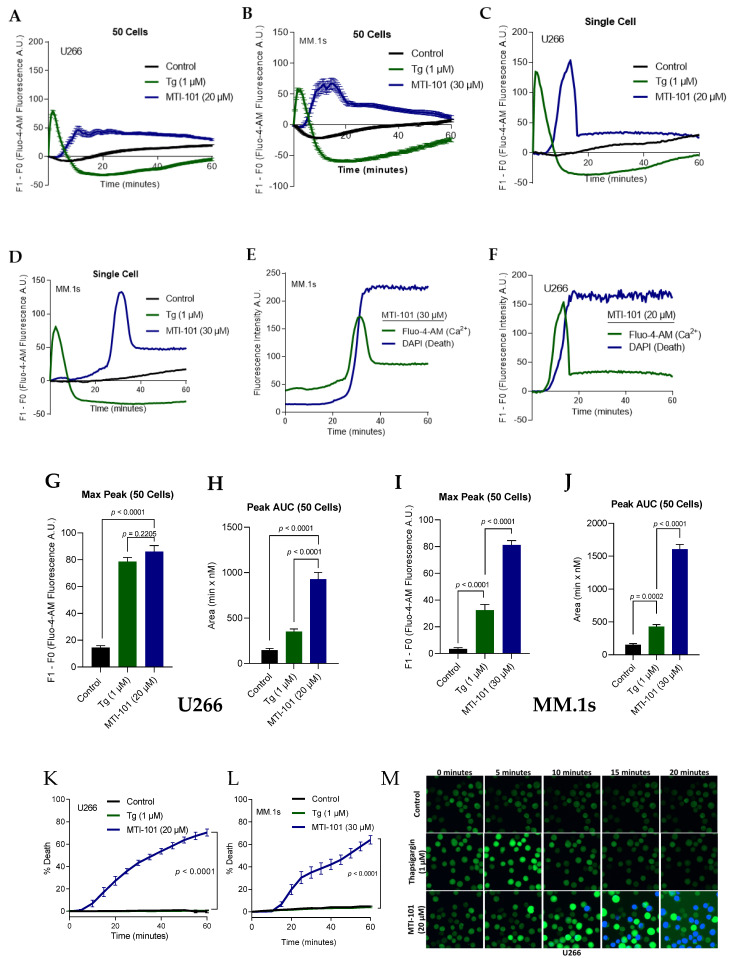
MTI-101 treatment Induces Sustained Ca^2+^ Influx and cell death in two independent MM cell lines. (**A**,**B**) The effect of MTI-101 was compared to thapsigargin (Tg) by measuring Ca^2+^ influx using Fluo-4-AM fluorescence intensity in U266 (**A**) and MM.1s (**B**) cell lines. The line graph shows the mean and standard error of the mean (SEM) of 50 cells fluorescence after subtracting the baseline over one hour. Cells were treated with MTI-101 (20 µM for U266, and 30 µM for MM.1s), Tg (1 µM), and vehicle control and imaged every 30 s. Shown is a representative experiment (*n* = 50 individual cells). (**C**,**D**) Shown is a single cell tracing for Ca^2+^ influx following treatment from figures A and B. The individual cells were chosen based on the median peak for Ca^2+^ influx for the respective treatment group. (**E**,**F**) Overlay of Fluo-4-AM and DAPI fluorescence in a single cell following MTI-101 treatment over one hour. (**G**,**H**) Mean maximum peak of Ca^2+^ influx in 50 cells in U266 and MM.1s cell lines. (**I**,**J**) The total levels of Ca^2+^ influx mediated by MTI-101, Tg, and vehicle control was measured by calculating the peak area under the curve (Peak AUC) of the 50 cells. (**K**,**L**) The effect of MTI-101 (20 µM) and Tg (1 µM) on cell death was measured in U266 and MM.1s cell lines by imaging the respective treatment group every 5 min for one hour. Cell death was determined by a threshold of DAPI fluorescence indicative of a dead cell. (**M**) Fluo-4-AM and DAPI pre-loaded U266 cells were used as indicators for Ca^2+^ uptake and cell death, respectively. Cells were imaged every 30 s at 10× using the BIOTEK Cytation 5 imager. All experiments were repeated three independent times, and shown is a representative experiment. Error bars represent SEM (*p* < 0.05 One-way ANOVA, inter-group comparison was done by Tukey’s multiple comparisons test).

We previously reported MTI-101 induced cell death in U266 and H929 MM cells through increased calcium flux leading to necrotic cell death [32]. To determine the involvement of SOCE pathway in MTI-101 induced cell death, we treated MM cells U266, MM.1s and the fibroblast stroma cell line HS-5 with MTI-101 and thapsigargin, and measured Ca^2+^ influx and cell death. MTI-101 treatment induced a delayed, and irreversible Ca^2+^ influx with peak accumulation occurring between 10 and 30 min. Thapsigargin induced a rapid, and robust Ca^2+^ influx in both U266 (Figure 1A,C,G,H,M) and MM.1s cell lines (Figure 1B,D,I,J) that peaked within the first five minutes of application. In the data presented in Figure 1C,D, we selected a cell representing the median Ca^2+^ peak of the 50 analyzed cells. Because time to peak varies in-between cells, this reshapes the curves when averaging the population (Figure 1A,B). MTI-101-induced cell death closely paralleled the time corresponding to the peak Ca^2+^ level (Figure 1E,F). HS-5 cells were treated with the same dose of MTI-101 (20 µM) and thapsigargin (1 µM) for the same treatment period (Supplementary Figure S1A–C). Interestingly, MTI-101 did not induce Ca^2+^ influx in HS-5 cells throughout the treatment period, while thapsigargin induced a rapid and robust Ca^2+^ influx, similar to MM cell lines. The lack of induction of Ca^2+^ influx correlated with the finding that MTI-101 did not induce cell death in HS-5 cells (Supplementary Figure S1D). Collectively, these data suggest that MTI-101 induced cell death in MM cells is correlated with a sustained, delayed, and irreversible Ca^2+^ influx, unlike thapsigargin which induces a rapid and reversible Ca^2+^ influx, that is not sufficient to induce immediate cell death. 

### 3.2. Extracellular Na^+^/K^+^/Ca^2+^ Contribution to MTI-101 Activity

**Figure 2:**
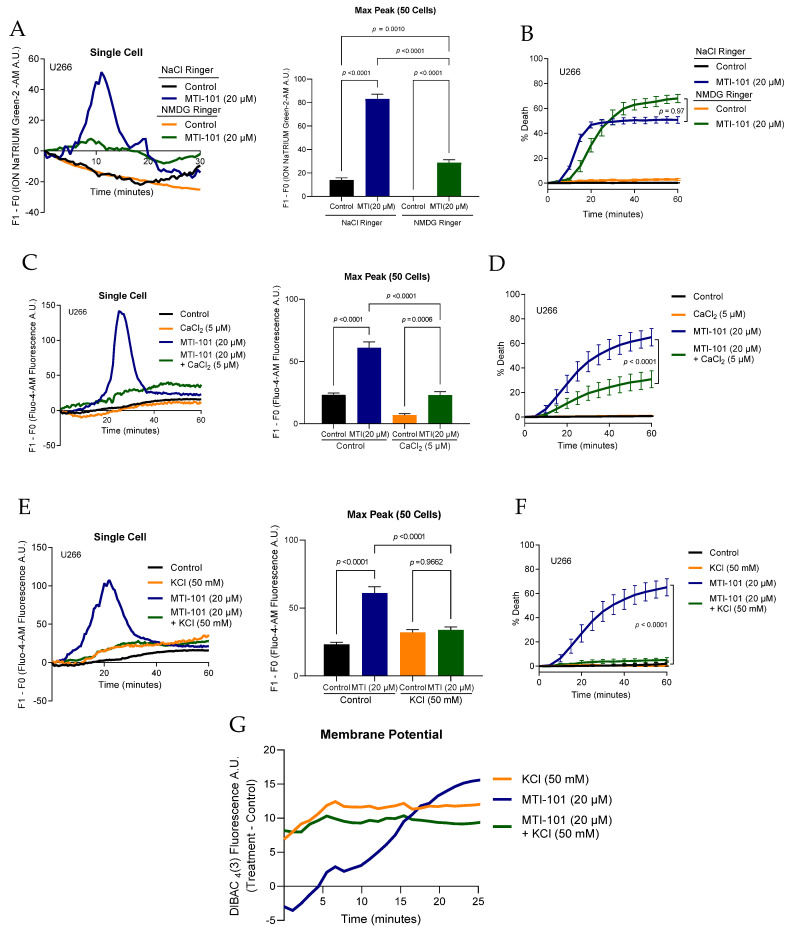
Extracellular Na^+^/Ca^2+^/K^+^ contribution to MTI-101 activity. (**A**) Shown is a single cell tracing for Na^+^ influx following treatment with MTI-101 (20 µM) in U266 cells. Na^+^ influx was measured using the Na^+^ indicator ION NaTRIUM Green-2 AM, and was imaged every 30 s. The individual cells were chosen based on the median peak for Na^+^ influx for the respective treatment group. Mean maximum peak of Na^+^ influx in 50 cells in U266. (**B**) The effect of MTI-101 (20 µM) on U266 cell death was measured by imaging the respective treatment groups in NaCl-ringer or NMDG-ringer solutions every five minutes for one hour. (**C**,**E**) Single cell tracing Ca^2+^ levels for U266 cells in 5 µM CaCl_2_, 50 mM KCl and physiological saline solutions with MTI-101 (20 µM). Cells were imaged every 30 s. Mean maximum peak of Ca^2+^ fluorescence in 50 cells. (**D**,**F**) The effect of MTI-101 (20 µM) on U266 cell death in 5 µM CaCl_2_, 50 mM KCl and physiological saline solutions. Cells were imaged every five minutes. (**G**) Fluorescence of DIBAC_4_(3) pre-loaded U266 cells treated with MTI-101 (20 µM) in either 50 mM KCl, or physiological saline solutions (3 mM KCl). Presented fluorescence data were measured by subtracting the mean control fluorescence of cells in 3 mM KCL PSS buffer from all other treatment groups. Cells were imaged every 30 s for 1 h. Error bars represent SEM (*p* < 0.05 One-way ANOVA, inter-group comparison was done by Tukey’s multiple comparisons test). All experiments were repeated three independent times, and shown is a representative experiment.

We next sought to determine the role of specific ions in mediating MTI-101-induced Ca^2+^ cytotoxicity. The Orai1 channel demonstrates high specificity for Ca^2+^ [35,36], whereas TRPC channel complexes are less specific for Ca^2+^ and additionally permeate both Na^+^ and K^+^ as well [21]. To learn more about the role of Na^+^ in MTI-101-mediated cytotoxicity, we first asked whether MTI-101 treatment induced Na^+^ influx. We addressed this question using the Na^+^ sensitive fluorescent dye ION NaTRIUM Green-2 AM, to measure the level of Na^+^ influx following MTI-101 treatment in both Na^+^-Ringer and NMDG-Ringer solutions (devoid of Na^+^ but contains the non-permeable monovalent cation N-methyl-d-glucamine or NMDG). MTI-101 treatment induced a peak in Na^+^ accumulation within ten minutes, similar to the pattern of MTI-101-induced Ca^2+^ entry. The Na^+^-sensitive fluorescence signal was significantly diminished but not completely abolished in cells bathed in Na^+^-free NMDG solution, suggesting a low degree of background fluorescence (Figure 2A). As shown in Figure 2B, replacing Na^+^ with NMDG was not sufficient to attenuate MTI-101-induced cell death. MTI-101 did, however, increase U266 cell permeability to Na^+^. Together, these data indicate that MTI-101 treatment induced activation of a Na^+^-permeable cationic channel, even though sodium influx per se does not play an apparent role in MTI-101-induced cell death.

To address the role of Ca^2+^ entry in MTI-101 induced cytotoxicity, we reduced extracellular CaCl_2_ from 2.5 mM to 5 µM CaCl_2_ and measured MTI-101 mediated Ca^2+^ accumulation and cell death. Decreasing external Ca^2+^ significantly reduced MTI-101 mediated Ca^2+^ influx (Figure 2C). Furthermore, decreasing extracellular Ca^2+^ significantly attenuated MTI-101 induced cell death by 34% in U266 cells (Figure 2D). Unlike Na^+^, extracellular Ca^2+^ contributes to MTI-101 mediated cell death. 

Calcium signaling in immune cells is modulated by K^+^ conductance that regulate the driving force for Ca^2+^ entry [37,38]. Plasma membrane potential is largely set by the K^+^ equilibrium across the membrane, and this in turn establishes the inward driving force for Ca^2+^. We sought to determine whether lowering the driving force for Ca^2+^ entry by depolarizing cells would modulate the potency of MTI-101. STIM1/Orai1 are continuously active in patch clamp experiments following store depletion by IP_3_ or ionomycin [15]. Further, when extracellular Ca^2+^ is depleted, and intact cells are treated with thapsigargin or cyclopiazonic acid, a large Ca^2+^ influx occurs when Ca^2+^ is re-added to the extracellular solution. This Ca^2+^ influx is attributed to the SOCE pathway and Ca^2+^ entry is driven by the negative membrane potential. However, in Jurkat T cells, when extracellular K^+^ levels are increased to 140 mM, the membrane potential collapses to 0 mV and Ca^2+^ entry is diminished due to the reduction in driving force. To test the effect of high extracellular K^+^ concentration on MTI-101-induced Ca^2+^ entry, we compared MTI-101-mediated Ca^2+^ entry among cells bathed in either physiological saline solution (3 mM KCl) or high K^+^ (50 mM KCl) saline solution. Elevated external K^+^ reduced Ca^2+^ accumulation by 55.6% (Figure 2E). Furthermore, high extracellular K^+^ concentration completely abolished MTI-101-induced cell death (Figure 2F). Moreover, we measured the membrane potential for the respective treatment groups in Figure 2 G using the membrane-potential sensitive dye DIBAC_4_(3) in U266 cells (Figure 2G). As expected, 50 mM KCl PSS resulted in membrane depolarization. MTI-101 treatment also increased dye fluorescence indicative of strong depolarization, likely caused by membrane depolarization due to Na^+^ influx through non-selective cation channels. Combining high K^+^ depolarization with MTI-101 failed to further depolarize the membrane potential of cells already depolarized by 50 mM KCl. Collectively, these data indicate that Ca^2+^ influx is the predominant driver for MTI-101-mediated cell death, and reducing Ca^2+^ influx either by reducing extracellular Ca^2+^, or reducing the driving force for Ca^2+^ diminishes MTI-101-mediated cell death. 

### 3.3. Pharmacological Inhibition of TRPC Channels Block MTI-101 Induced Ca^2+^/Na^+^ Influx and Cell Death in MM Cell Lines and Inhibition of CRAC Channels Inhibits MTI-101 Induced Ca^2+^ Influx and Cell Death

**Figure 3:**
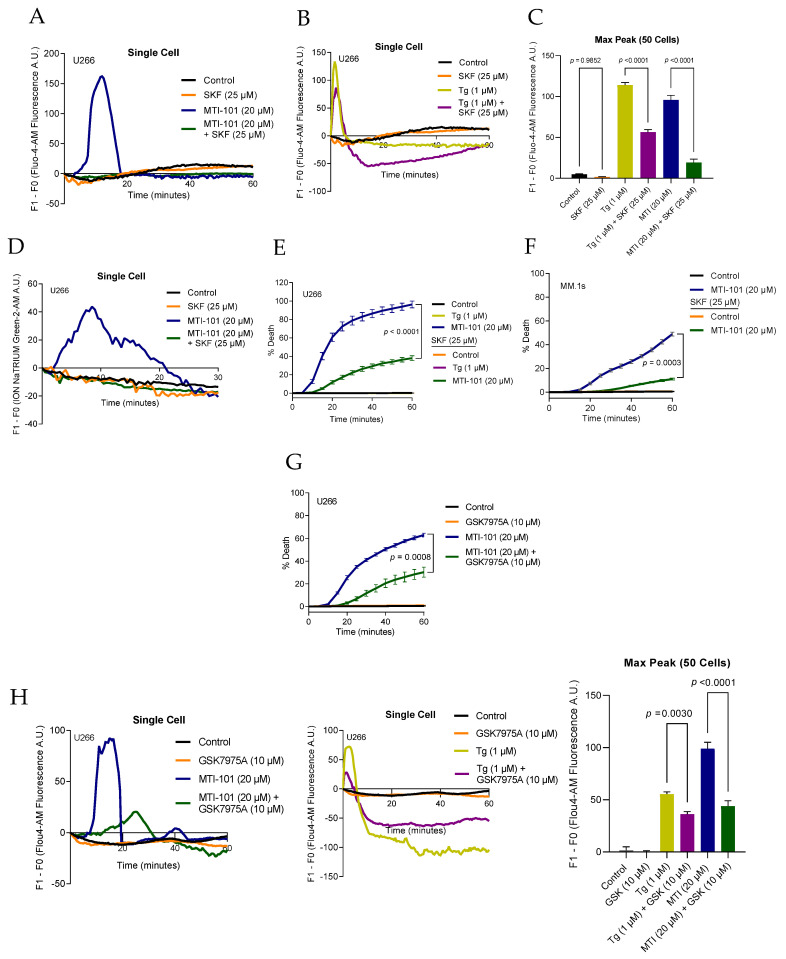
Pharmacological inhibition of TRPC channels blocks MTI-101 induced Ca^2+^/Na^+^ influx and cell death in MM cell lines and inhibition of CRAC channels inhibits MTI-101 induced Ca^2+^ influx and cell death. (**A**,**B**) The effect of blocking non-specific cation channels was tested using the inhibitor SKF96365 (25 µM) in combination with MTI-101 (20 µM) and Tg (1 µM) on U266 cell line. Ca^2+^ influx fluorescence signal was measured every 30 s in a single cell. The individual cells were chosen based on the median peak for Ca^2+^ influx for the respective treatment group. (**C**) Mean maximum peak of Ca^2+^ influx in 50 cells in U266 cell line. (**D**) Single cell tracing of Na^+^ influx in cells pre-treated with SKF96365 (25 µM) followed by MTI-101 (20 µM) treatment. Cells were imaged every 30 s. The individual cells were chosen based on the median peak for Na^+^ influx for the respective treatment group. (**E**,**F**) The effect of SKF96365 (25 µM) and MTI-101 (20 µM) on cell death was measured in U266 and MM.1s cell lines. Cell death was measured every five minutes. (**G**) The effect of blocking CRAC channels was determined using the inhibitor GSK7975A (10 µM) in combination with MTI-101 (20 µM) on U266 cell death. Cell death was measured every five minutes. (**H**) Single cell tracing Ca^2+^ levels for GSK7975A (10 µM) pre-treated U266 cells followed by MTI-101 (20 µM) or Tg (1 µM). Cells were imaged every 30 s. Mean maximum peak of Ca^2+^ fluorescence in 50 cells. Error bars represent SEM (*p* < 0.05 One-way ANOVA, inter-group comparison was done by Tukey’s multiple comparisons test).

Even though Na^+^ influx was not responsible for cell death, experimental data indicated that MTI-101 treatment resulted in influx of both Na^+^ and Ca^2+^, prompting us to determine the role of TRPC channels in mediating the influx of these ions. We initially sought to use pharmacological tools to delineate the role of TRPC channels in mediating MTI-101 induced cell death. SKF96365 is a somewhat non-specific inhibitor of receptor-mediated Ca^2+^ entry pathways (RMCE) and SOCE [39,40]. We pre-treated U266 cells with SKF96365 (25 µM) for 1 h and measured Ca^2+^ influx in the presence of MTI-101 and Tg, respectively (Figure 3A,B). Cells pre-treated with SKF96365 showed a significant reduction in Ca^2+^ entry when stimulated with either MTI-101 (Figure 3A) or Tg (Figure 3B). SKF96365 reduced peak Ca^2+^ accumulation evoked by MTI-101 by 80%, and decreased the peak Tg-induced Ca^2+^ response by 51% (Figure 3C). Because SFK96365 treatment blocked the Tg-induced Ca^2+^ response, these data suggest that SKF9635 likely also blocks CRAC channels and therefore precludes a clear discrimination between CRAC and TRPC-mediated Ca^2+^ influx in MM cells. 

To further test the idea that the MTI-101-mediated ion influx might be carried at least in part by TRPC channels that can conduct both Na^+^ and Ca^2+^, we asked whether SKF96365 could inhibit MTI-101-mediated Na^+^ influx. SKF96365-pretreatment significantly reduced Na^+^ accumulation in U266 cells by 29% measured by average maximum Na^+^ peak of 50 cells (Figure 3D) and decreased MTI-101-induced cell death in both U266 and MM.1 cells by 58% and 38%, respectively (Figure 3E,F). These data suggest that MTI-101 activates a non-specific cation channel that contributes to MM cell death and can be modulated by pre-treatment with SKF96365. To address the role of CRAC channels on MTI-101-induced cell death and Ca^2+^ influx, we pre-treated U266 cells with CRAC specific inhibitor GSK7975A (Figure 3G). GSK7975A (10 µM) attenuated MTI-101-induced cell death by 32%. GSK7975A attenuated thapsigargin-induced Ca^2+^ influx, indicating the specificity of the drug towards CRAC activity, and significantly reduced MTI-101 induced Ca^2+^ influx (Figure 3H). Taken together, the CRAC channels inhibitor GSK7975A attenuates MTI-101 induced Ca^2+^ influx and cell death.

### 3.4. TRPC4/5 Channel Contributes to MTI-101 Induced Ca^2+^/Na^+^ Influx and Cell Death

**Figure 4:**
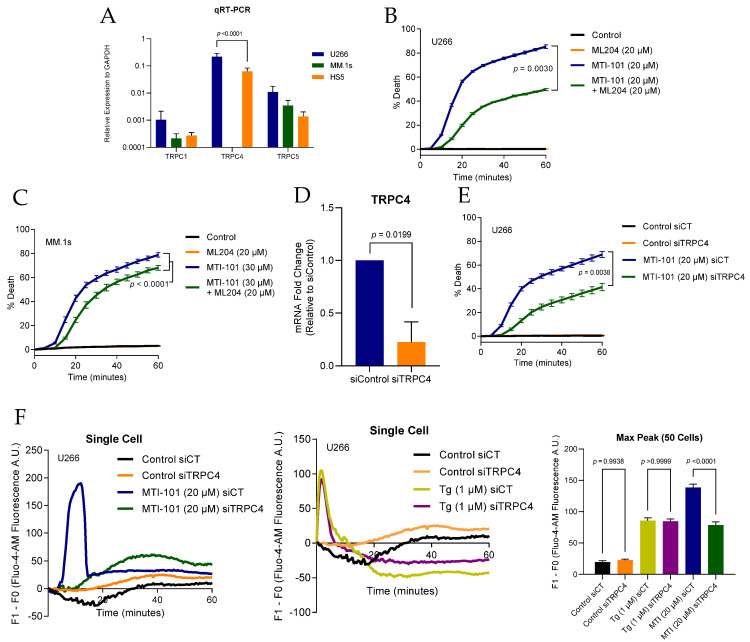
TRPC4 channel contributes to MTI-101 induced Ca^2+^/Na^+^ influx and cell death. (**A**) TRPC1, TRPC4, and TRPC5 mRNA relative expression levels in U266, MM.1s, and HS-5 cell lines. Abundance of expression is shown for the respective mRNA compared to GAPDH in each cell line. Experiments were done in triplicates three independent times. Shown is the mean of the three experiments. (**B**,**C**) The effect of ML204 (20 μM) pre-treatment on U266 and MM.1s cell death with MTI-101 (20 μM and 30 μM respectively). Error bars represent SEM, experiments were done three independent times, shown is a representative single experiment (*p* < 0.05 One-way ANOVA). (**D**) TRPC4 mRNA fold change expression in U266 with siTRPC4 relative to U266 with siControl is shown. SEM of three independent experiments is shown (*p* < 0.05 student *t*-test). (**E**) Effect of knocking down TRPC4 in U266 cell death by MTI-101 (20 μM). Experiments were done three independent times, and shown is a single representative experiment (*p* < 0.05 One-way ANOVA). (**F**) Single-cell tracing for Ca^2+^ influx in Fluo-4-AM preloaded U266 cells with siTRPC4 and siControl. Cells were treated with MTI-101 (20 μM) and Tg (1 μM). Cells were imaged every 30 s for one hour.

We previously created a MM cell line resistant to HYD-1 (linear MTI-101) H929-60, and reported a down regulation of multiple genes contributing to Ca^2+^ entry pathways, including TRPC1, ATP2A3, PLC-β, ITPR3, and TRPM7 [32]. Further, we reduced the expression of TRPC1 via shRNA and noted a reduction in MTI-101 induced cell death. Moreover, the broad-spectrum IP_3_ and TRPC channel inhibitor 2-APB significantly inhibited MTI-101 induced cell death in relapsed MM patients’ specimens, U266 and H929 cell lines [32]. The TRPC family is comprised of seven members, and functional TRPC1-containing channels are formed by heterodimeric and heterotetrameric arrangements of subunits (e.g., TRPC1/4, TRPC1/5, TRPC1/4/5) [22,24,41]. To learn more about the possible components of TRPC channels expressed in MM cells, we measured the expression levels of TRPC1, TRPC4 and TRPC5 in MM cell lines by quantitative real-time PCR in U266 and MM.1s cell lines. Interestingly, TRPC1 and TRPC5 were both abundantly expressed in U266, MM.1s and HS-5 while TRPC4 was only expressed in U266 and HS-5 (Figure 4A). To determine the role of TRPC4 in MTI-101 induced cell death, we pretreated U266 and MM.1s cells with ML204 (20 µM), a selective inhibitor of TRPC4 and TRPC5 channels, with no reported selectivity towards other TRP family members [42]. Pre-treated U266 cells showed a 49% significant attenuation to MTI-101-induced cell death, with only 22% attenuation in MM.1s cells (Figure 4B,C). These data suggest that pharmacological inhibition of TRPC4 and TRPC5 moderately reduced MTI-101 cytotoxicity. The efficacy of ML204 may have been confounded by the heterogeneity of TRPC channel complexes. 

To further address the role of TRPC4, small interfering-RNA (siRNA) was used to reduce the expression of TRPC4 (Figure 4D). Reducing the expression of TRPC4 significantly inhibited MTI-101 induced cell death by 50%, supporting the role of TRPC4 in MTI-101-mediated cell death (Figure 4E). Interestingly, Ca^2+^ influx was significantly reduced by 57% in MTI-101 treated TRPC4 knockdown cells, but was not attenuated in the Tg-treated cells (Figure 4F), indicating that both SOCE and RMCE pathways may contribute to Ca^2+^ influx independently. 

**Figure 5:**
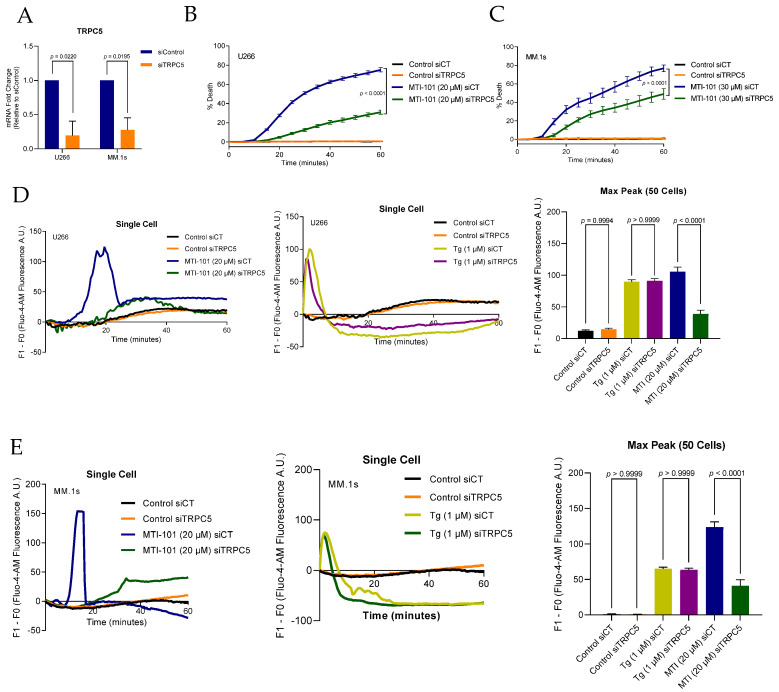
TRPC5 channel contributes to MTI-101 induced Ca^2+^/Na^+^ influx and cell death. (**A**) TRPC5 mRNA fold change expression in U266 and MM.1s with siTRPC5 relative to siControl cell lines. SEM of three independent experiments is shown (*p* < 0.05 student *t*-test). (**B**,**C**) Effect of knocking down TRPC5 in U266 and MM.1s on cell death mediated by MTI-101 (20 µM and 30 µM respectively). Experiments were done three independent times, and shown is a single representative experiment (*p* < 0.05 One-way ANOVA). (**D**,**E**) Single cell tracing for Ca^2+^ influx in Fluo-4-AM loaded U266 and MM1.s cells with siTRPC5 and siControl. Cells were treated with MTI-101 (20 μM) and Tg (1 μM). Cells imaged every 30 s for one hour. Mean maximum peak of Ca^2+^ influx in 50 cells in U266 and MM.1s cell lines. Error bars represent SEM (*p* < 0.05 One-way ANOVA, inter-group comparison was done by Tukey’s multiple comparisons test *p* < 0.0001).

To test whether TRPC5 expression is a determinant in MTI-101 induced Ca^2+^ influx and cell death, we used siRNA to knockdown TRPC5 expression in U266 and MM.1s cell lines (Figure 5A). Reducing TRPC5 expression attenuated MTI-101 induced cell death by 60% and 36% in U266 and MM.1s respectively (Figure 5B,C). Furthermore, cells with reduced expression of TRPC5 showed a significant reduction in MTI-101 induced Ca^2+^ influx by 37% and 33% in both cell lines, respectively, similar to what was observed by reducing TRPC4 expression (Figure 5D,E). Finally, TRPC5 knockdown had no discernable effect on peak Tg-induced Ca^2+^ influx levels (Figure 5D,E). Taken together, these data indicate that TRPC4 and TRPC5 significantly contribute to the MTI-101 induced Ca^2+^ influx and cell death, delineating a role of TRPC channels in MTI-101-mediated Ca^2+^ influx in parallel to the canonical SOCE pathway.

### 3.5. MTI-101 Treatment Induces TRPC1 Trafficking to the Plasma Membrane and Formation of STIM1/TRPC1 Complex

**Figure 6:**
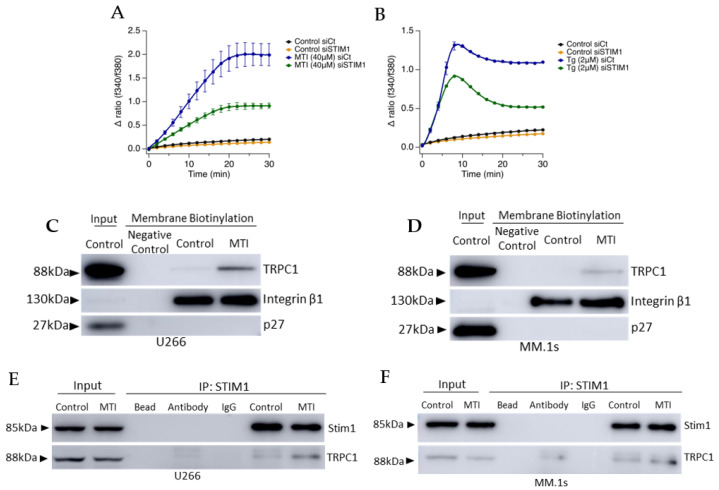
MTI-101 treatment induces TRPC1 trafficking and formation of STIM1/TRPC1 complex. (**A**,**B**) Ca^2+^ accumulation in populations of approximately 60,000 U266 cells loaded with Fura-2AM with STIM1 siRNA or siControl. Cells were treated with either MTI-101 (40 μM) or Tg (2 μM). Cells were imaged every 3 s for 30 min. Error bars show SEM. (**C**,**D**) Detection of TRPC1 in the biotinylated membrane proteins in U266 and MM.1s cell lines following treatment with MTI-101 (20 µM and 30 µM, respectively) for 10 min. P27 used as cytoplasmic control and Integrin β1 is a plasma membrane control. (**E**,**F**) Co-localization detection of SOCE pathway proteins was done by co-immunoprecipitation of STIM1 following MTI-101 treatment for 10 min in U266 and MM.1s cells (20 µM and 25 µM, respectively). Experiments were done three independent times.

TRPC1 appears to contribute to the sustained Ca^2+^ influx following MTI-101-induced signaling in parallel with the canonical SOCE pathway, as has been previously reported [43]. To determine whether STIM1 was necessary for MTI-101-mediated Ca^2+^ entry, we examined the effect of knocking down STIM1 expression by siRNA on Ca^2+^ signaling (Figure 6A,B). Reducing STIM1 expression decreased the Tg-evoked rise in store operated Ca^2+^ entry by 40%, and reduced MTI-101-mediated Ca^2+^ entry by 64%. I_SOC_ currents mediated by STIM1/TRPC1 complex occurs following the trafficking of TRPC1 to the plasma membrane following the initial activation of STIM1/Orai1 [44]. To determine whether MTI-101 treatment induces trafficking of TRPC1 to the plasma membrane, we treated U266 and MM.1s cells with MTI-101 (20 µM and 25 µM, respectively) for 10 min followed by a membrane biotinylation of surface proteins (Figure 6C,D). Cells treated with MTI-101 showed an increased level of TRPC1 in comparison to control. We utilized p27 as a cytoplasmic/nuclear control of protein to ensure increased levels of TRPC1 were not due to MTI-101 induced increases of the permeability of the chemical probe and increased detection of cytoplasmic protein. As shown in Figure 6E,F treatment with MTI-101 induces STIM1/TRPC1 complex following 10 minutes of drug treatment in U266 and MM1.s cells. Together, these findings support the hypothesis that MTI-101 activity is dependent on STIM1 expression, TRPC1 trafficking and incorporation into the plasma membrane, and a STIM1/TRPC1 complex formation. 

### 3.6. MTI-101 Induces TRPC1 Truncation by Calpain Activation

**Figure 7:**
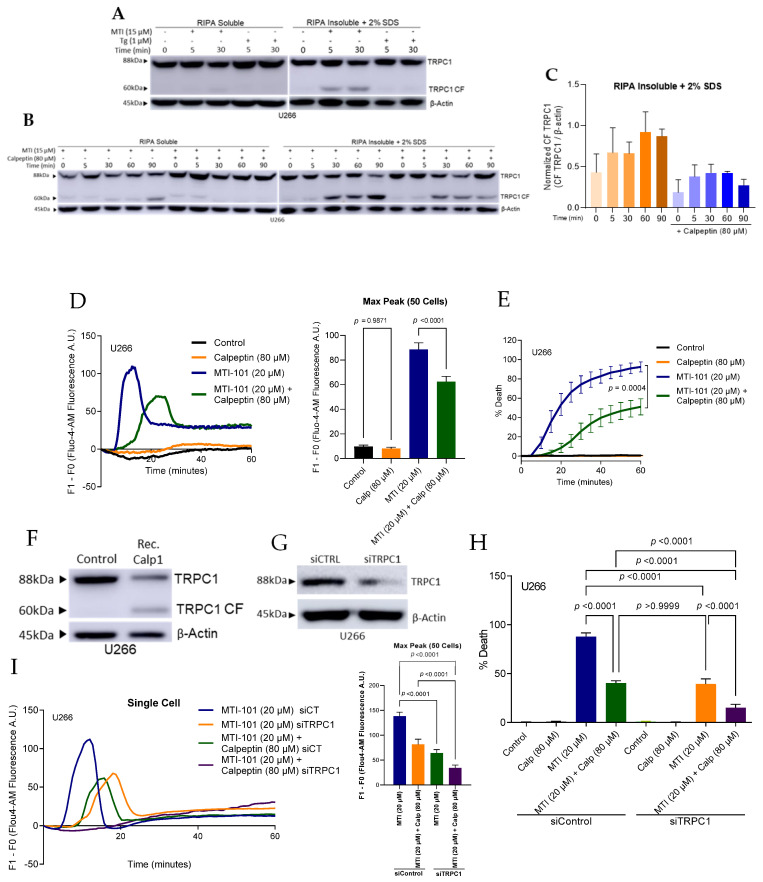
MTI-101 induces TRPC1 Truncation by Calpain Activation. (**A**) U266 cells treated with MTI-101 (15 μM) and Tg (1 μM) for 5 and 30 min. TRPC1 cleaved fraction was detected at ≈60 kDa only with MTI-101 treatment in RIPA insoluble cell fraction. (**B**,**C**) U266 cells pretreated with calpeptin (80µM) for one hour, followed by treatment with MTI-101 (15 µM) for 0, 5, 30, 60, 90 min. TRPC1 cleaved fractions was detected in the calpeptin-free group. Quantification of the cleaved fraction TRPC1 normalized to β-actin. (**D**) Single cell Ca^2+^ tracing for Fluo-4-AM pre-loaded U266 cells pretreated with calpeptin (80 µM) for one hour. Cells were imaged every 30 s for one hour. Mean maximum peak of Ca^2+^ influx in 50 cells in U266 cells. Error bars represent SEM (*p* < 0.05 One-way ANOVA, inter-group comparison was done by Tukey’s multiple comparisons test *p* < 0.05). (**E**) The effect of calpeptin (80 µM) pretreatment on MTI-101 induced cell death in U266 cells. Experiments were done in quadruplicates and repeated three independent times. Error bars represent SEM (*p* < 0.05 One-way ANOVA, inter-group comparison was done by Tukey’s multiple comparisons test *p* < 0.05). (**F**) CaCl_2_ activated calpain I induced TRPC1 cleaved fraction at ≈60kDa in U266 cells. (**G**) Western blot analysis for TRPC1 expression levels in U266 cells. (**H**) Effect of knocking down TRPC1 in U266 cells on cell death mediated by MTI-101 (20 µM) at 1 h (*p* < 0.05 One-way ANOVA). (**I**) Single cell tracing for Ca^2+^ influx in Fluo-4-AM loaded U266 with siTRPC1 and siControl. Cells were treated with MTI-101 (20 μM) with or without one-hour pre-treatment with calpeptin (80 μM). Cells imaged every 30 s for one-hour. Mean maximum peak of Ca^2+^ influx in 50 cells in U266 cell line. Error bars represent SEM (*p* < 0.05 One-way ANOVA, inter-group comparison was done by Tukey’s multiple comparisons test *p* < 0.0001).

Kaczmarek and colleagues reported that the Ca^2+^ activated proteases calpain I/II cleaves and further activate TRPC5 in neuronal cells in the presence of semaphorin 3A [45]. Further, they reported that the calpain inhibitor calpeptin blocks the cleavage and attenuates downstream Ca^2+^ currents. In addition, the Ca^2+^ influx through TRPC1 activity is reported to activate calpains [46]. The additional impact provided by MTI-101 activity is the absence of a turn off switch to the Ca^2+^ currents, resulting in sustained Ca^2+^ entry and leading to cell death. To determine the potential role of calpain I/II in MTI-101 effects, we treated cells with MTI-101 (15 µM) and Tg (1 µM) and analyzed the RIPA insoluble cellular compartment via Western blot analysis (Figure 7A). Interestingly, we noticed a cleaved TRPC1 fraction at 60 kD molecular weight only with MTI-101 treatment. Further, we noticed decreased levels of TRPC1 cleaved fractions when we pre-treated cells with calpeptin (80 µM) in U266 and MM.1s cells (Figure 7B,C, and Supplementary Figure S2A,B). Moreover, calpeptin pre-treatment significantly reduced Ca^2+^ influx and MTI-101-induced cell death (Figure 7 D,E). To assess whether TRPC1 cleavage is calpain I -dependent, we treated sonicated U266 and HS-5 stroma lysates with recombinant calpain I (Figure 7F, and Supplementary Figure S2C). Recombinant calpain I cleaved TRPC1 at approximately the same molecular weight as the downstream effect of MTI-101 treatment. Furthermore, pre-treatment with the calpain inhibitor calpeptin blocked the cleavage of TRPC1 (Supplementary Figure S2D). We found that TRPC1 in HS-5 stroma cells are prone to cleavage by active calpain I, while MTI-101 did not show any associated TRPC1 cleaved fraction (Supplementary Figure S2E). Previously we reported that decreasing TRPC1 protected from MTI-101 induced cell death[32]. To test the effect of TRPC1 on MTI-101-induced Ca^2+^ influx, we used siRNA to knockdown TRPC1 expression in U266 and MM.1s cell lines (Figure 7F and Supplementary Figure S2F).Reducing TRPC1 expression attenuated MTI-101 induced cell death by 48% and 20% in U266 and MM.1s respectively, furthermore, calpain pre-treatment in cells with reduced TRPC1 expression attenuated MTI-101 effect by 72% and 76% in both cell line respectively (Figure 7G and Supplementary Figure S2G). Reducing TRPC1 showed a significant reduction in MTI-101 induced Ca^2+^ influx in both cell lines, and calpain pre-treatment contributed to increased reduction in Ca^2+^ levels (Figure 7H and Supplementary Figure S2H). In aggregate, these data suggest that calpain I activation following MTI-101 treatment contributes to the sustained Ca^2+^ influx, and is potentially a feed forward loop for activation of TRPC containing channels following treatment with MTI-101 in MM cells. 

### 3.7. Members of the SOCE Pathway Correlates with Poor Patient Outcomes

**Figure 8:**
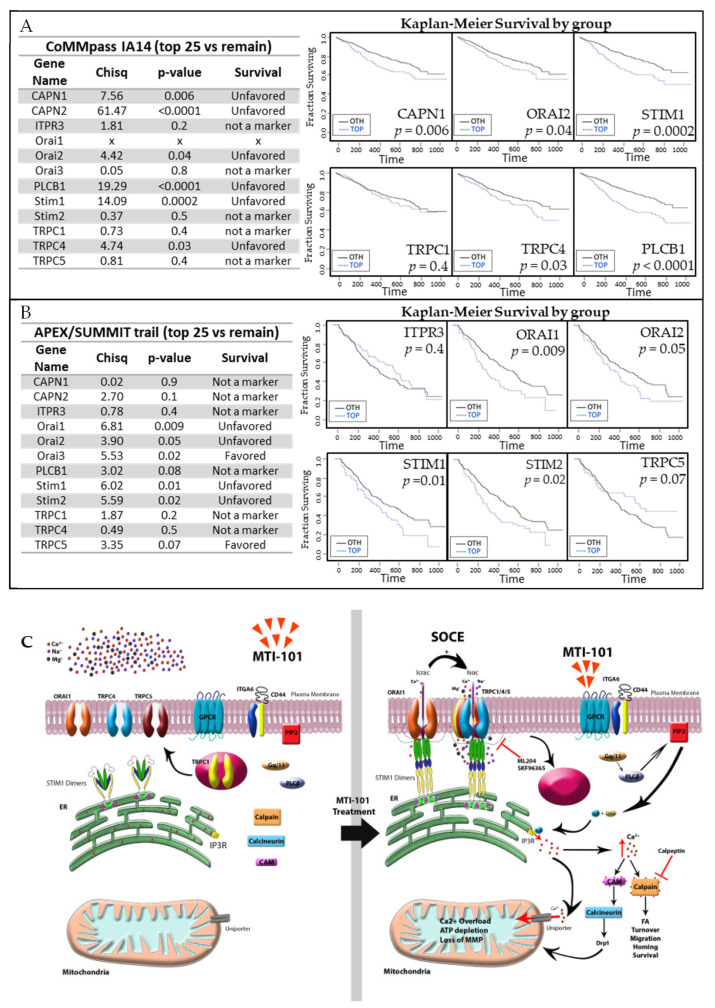
Members of the SOCE pathway correlates with poor patient outcomes. (**A**) Overall survival analysis for selected components of the SOCE pathways for newly diagnosed MM patients from the CoMMpass study. (**B**) Overall survival analysis for selected components of the SOCE pathway for relapsed MM patients in the APEX/SUMMIT trail. p-value was determined by log-rank test; (x) indicates expression data not available. Time is shown in days. (**C**) A working model for MTI-101 mechanism of action.

Aside from the SOCE role in replenishing ER/SR Ca^2+^ stores, the extracellular Ca^2+^ influx through TRPC regulates multiple Ca^2+^-dependent gene expressions patterns (ex. NFAT pathway) [47]. Increased TRPC1 expression has been reported to contribute to pancreatic cancer cell motility, breast cancer, non-small cell lung cancer, colon cancer, and glioblastoma multiforme proliferation and migration [48,49,50,51,52]. Further, we previously discussed the role of Ca^2+^ homeostasis in cancer progression. Interestingly, this class of compounds is more active in CD138 cells isolated from MM patients that have relapsed compared to newly diagnosed patients [30,32]. These data suggest that determinants of MTI-101 sensitivity including the SOCE pathway maybe upregulated in aggressive MM disease. Thus, we sought to determine whether expression of the components of the SOCE pathway correlate with aggressive disease. To address this question, we used two publically available gene expression datasets with paired patient survival data. The COMPASS data set is multi-site trial initiated by MMRF [53]. Expression profiling was based on newly diagnosed specimens using RNA-SEQ as the platform (Figure 8A). The APEX/SUMMIT data was a multi-site international trial for patients enrolled in Phase II and Phase III clinical trials of Bortezomib [34]. Thus, these patients were relapsing on current therapy prior to proteasome related regimens. The expression profiling platform was GeneArray 3000 Affymetrix for this study. As shown in Figure 8A,B, multiple components of the SOCE pathway were poor or unfavorable prognostic markers in both studies. Interestingly, TRPC components were not as strong predictors compared to STIM1. However, these data do not rule out that a readout of activity such as plasma membrane localization of TRPC1 may be required for prediction of disease outcome. Future studies will address the role of membrane localized TRPC1 as a prognostic indicator for MM progression. In Figure 8C, we show a working model for the mechanism of action of MTI-101 based on the current data presented in this manuscript and previously reported data [30,31,32]. 

## 4. Discussion

Ca^2+^ entry through SOCE regulates multiple genetic pathways, where Ca^2+^ signals through CRAC currents activate the NFAT and NFkB pathways; Ca^2+^ currents through TRPC1 and the I_SOC_ complex predominantly activate the NFkB pathway [47,54,55]. Although TRPC1 expression contributes to various tumor outcomes, its over expression is predominantly and most consistently correlated with poor cancer outcomes in lung, pancreatic, breast, glioblastoma multiforme, and colon cancers [24,48,49,50,51,52]. In this study, we show that RNA expression of components of the SOCE pathway correlates with poor survival in newly diagnosed MM patients when examining the expression levels in 1150 patients using the CoMMpass dataset and 669 relapsed patients in the APEX/SUMMIT trial. Based on these data it is intriguing to speculate that increased capacity to respond to the RMCE/SOCE pathway is required for aggressive disease and potentially contributes to the emergence of drug refractory disease. Interestingly, at the RNA level STIM1/ORAI was a stronger prognostic indicator compared to components of TRPC. However, considering our data indicating that TRPC1 needs to be trafficked to the cell membrane, it is feasible that protein localization rather than absolute expression levels might be a determinant of aggressive disease. This signature may lead to strategies to pharmacologically block this pathway or conversely, represents an Achilles heel of aggressive disease having increased susceptibility to cell death via Ca^2+^ overload. With that in mind, targeting Ca^2+^ homeostasis, and more specifically the SOCE pathway presents a novel pathway for the treatment of MM. 

Our findings indicate that the MTI-101 mechanism of action is different from the standard blocker of the SERCA pump thapsigargin. While thapsigargin inhibits SERCA function causing rapid depletion of the ER Ca^2+^ stores, MTI-101 treatment induces a more delayed and sustained Ca^2+^ entry. This difference could be attributed to the I_CRAC_ currents through STIM1 coupling with Orai1 activity, while MTI-101 is mostly dependents on I_SOC_ currents through STIM1/TRPC1/4/5 activity, but not completely independent of STIM1/Orai1 activity. Baudel and colleagues previously reviewed the role of SOCE in vascular smooth muscles (VSMCs) contractility, where cells isolated from mice with Orai1-/- were capable of activating the SOCE pathway via STIM1 and TRPC1 only [56,57]. Furthermore, Romani et al. reported that, in arterial myocytes, Orai1 expression levels were inherently low in the contractile phenotype, yet maintained SOCE activity, while the proliferative phenotype showed increased expression of Orai1, STIM1, and TRPC1 [58]. In contrast, it has been reported that in salivary glands, I_SOC_ currents mediated through STIM1 and, TRPC1 interaction is dependent on prior activation of STIM1/Orai1, and the knockdown of Orai1 abolishes TRPC1 activation [59]. 

Owing to this diversity in TRPC1 mode of activation, it is conceivable that MTI-101 activity could be dependent on coupling STIM1 and TRPCs in MM cells. Consistent with this notion, we observed that MTI-101-induced cell death is partially dependent on the expression of TRPC1/4/5, suggesting that they participate in the complex formation with STIM1. We further report that, in an inactive state, and in the absence of MTI-101, we failed to detect TRPC1 at the plasma membrane, and the activation of the SOCE pathway by MTI-101 allowed TRPC1 trafficking and insertion into the plasma membrane. TRPC1 recycling to and from the plasma membrane following SOCE activation has been reported previously by De Souza and colleagues [60]. They reported that the TRPC1 fast recycling is dependent on trafficking to the plasma membrane by Rab4 and the internalization into Rab5-containing endosomes is an ARF6-dependent pathway. Future studies will determine the mechanism underpinning MTI-101-induced trafficking of TRPC1 to the plasma membrane. We propose that MTI-101 is a unique tool for the delineation of TRPC1 membrane trafficking.

The Ca^2+^-activated protease calpain 1 is associated with poor survival in MM patients in the COMPASS dataset. Kaczmarek et al. reported that the pharmacological inhibition of calpain I/II inhibited TRPC5 [45]; further, Verheijden and colleagues reported that TRPC6 contributes to calpain I activation, and pharmacologically inhibiting calpain I or genetically knocking it down abrogated TRPC6 effects on podocyte injury in the kidneys [61]. Other reports indicated that Ca^2+^ entry through active TRPC1 activates calpain I in neurons and regulates axon outgrowth [46]. Our findings indicated that MTI-101 treatment induces cleavage of TRPC1 and that TRPC1 is a substrate for recombinant calpain I. In addition, pharmacological inhibition of calpains blocked MTI-101-induced calcium influx and cell death. Based on these data it is attractive to reason that the calpain-dependent feed-forward loop is due to cleavage of TRPC1 either via induction of a higher conductance or open probability of the channel. Further studies are required to determine the functional consequence of TRPC1 cleavage on MTI-101-induced calcium and sodium entry. Cleavage of TRPC1 was targeted in cells sensitive to MTI-101, while cleavage was not observed in the stroma cell line HS-5. However, Ca^2+^-activated recombinant calpain I was able to cleave TRPC1 from HS-5 derived lysates. These finding correlate with failure of MTI-101 to evoke a calcium response in HS-5 stroma cells. Again, more studies are required to fully understand the mechanism underpinning the MTI-101-induced trafficking of TRPC1 to the plasma membrane, and its continuous activation leading to the sustained Ca^2+^ influx and cell death. 

## 5. Conclusions

In summary, MTI-101 is a novel drug that leverages one of the cancer cells’ main survival mechanisms, the augmented Ca^2+^ circuitry. Future studies will define the dependency of TRPC1 for survival in the bone-marrow microenvironment and whether this signaling pathway is required for growth, cell adhesion-mediated drug resistance (CAM-DR), or soluble mediate drug resistance (SM-DR). Our data support a unique mechanism of Ca^2+^-induced necrotic cell death and the targeting of store-operated Ca^2+^ entry pathways for the treatment of multiple myeloma. 

## Supplementary Materials

The following are available online at https://www.mdpi.com/article/10.3390/cells10061490/s1, Figure S1: Stroma cells are not sensitive to MTI-101-induced Ca^2+^ flux and cell death, Figure S2: MTI-101 induces TRPC1 Truncation by Calpain Activation.
