# Supplementary material for: Transient Receptor Potential C 1/4/5 Is a Determinant of MTI-101 Induced Calcium Influx and Cell Death in Multiple Myeloma
Source: Cells. 2021 Jun 13;10(6):1490. doi: 10.3390/cells10061490 (PMC8231892; doi:10.3390/cells10061490)
Supplement: Supplementary file 1 [file cells-10-01490-s001.zip › cells-1208373-supplementary.pdf]

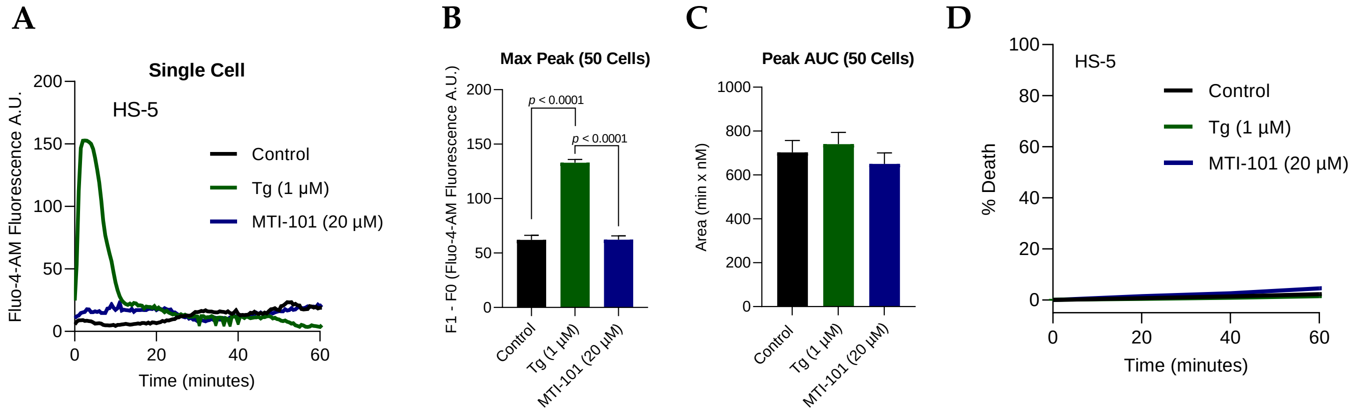

Figure S1. Stroma cells are not sensitive to MTI-101-induced Ca2+ flux and cell death: A and B) The effect of MTI-101 was compared to thapsigargin (Tg) by measuring Ca^{2+} influx using Fluo-4-AM fluorescence intensity in HS-5 cell line. The line graph shows a single cell tracing for Ca^{2+} influx following treatment with MTI-101 (20 µM) and Tg (1 µM). The individual cells were chosen based on the median peak for Ca^{2+} influx for the respective treatment group. Cells were imaged every 30 seconds. **B)** Mean maximum peak of Ca^{2+} influx in 50 cells in HS-5 cell line. Error bars represent SEM ($p < 0.05$ One-way ANOVA, inter-group comparison was done by Tukey's multiple comparisons test $p < 0.05$). **C)** The total levels of Ca^{2+} influx mediated by MTI-101, Tg, and vehicle control was measured by calculating the peak area under the curve (Peak AUC) of the 50 cells ($p < 0.05$ One-way ANOVA, inter-group comparison was done by Tukey's multiple comparisons test $p < 0.05$). **D)** The effect of MTI-101 (20µM) and Tg (1µM) on cell death was measured in HS-5 cells by imaging the respective treatment group every 5 minutes for one hour. Cell death was determined by a threshold of DAPI fluorescence indicative of a dead cell. Error bars represent SEM ($p < 0.05$ one-way ANOVA).

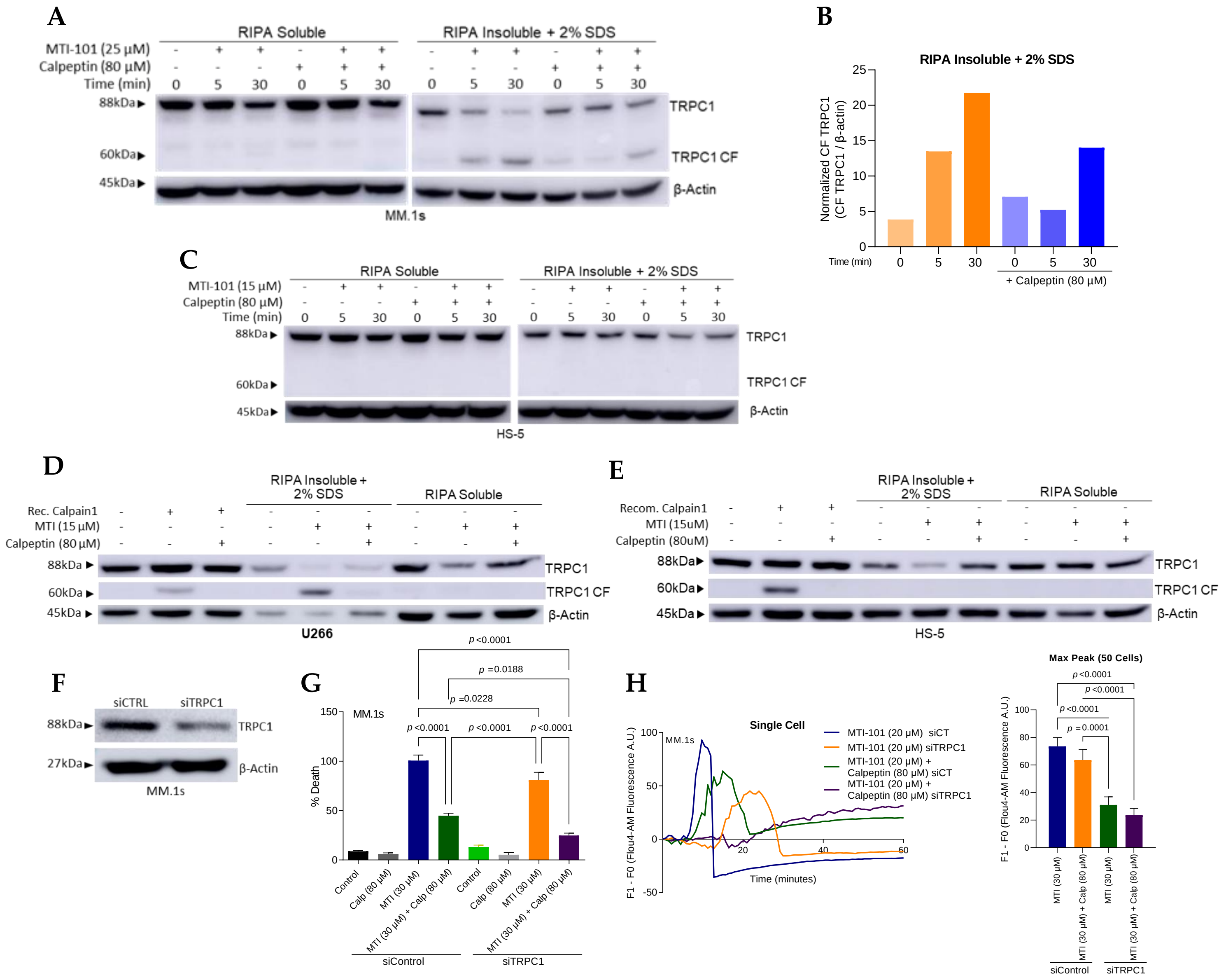

Figure S2: MTI-101 induces TRPC1 Truncation by Calpain Activation: A and B) MM.1s cells pretreated with calpeptin (80 µM) for one hour, followed by treatment with MTI-101 (25 µM) for 0, 5, 30 minutes. TRPC1 cleaved fractions was detected at ≈60 kDa in the calpeptin-free group in the RIPA insoluble compartment. Quantification of the cleaved fraction TRPC1 normalized to β-actin. **C)** MTI-101 (15 µM) does not induce TRPC1 cleavage in HS-5 cells in both RIPA Soluble and Insoluble compartments. **D)** $CaCl_2$ activated calpain I induced TRPC1 cleaved fraction at ≈60kDa in U266 cells following 1-hour incubation with the recombinant protein. Pre-treating cells with calpeptin (80 µM) for 1 hour abrogated TRPC1 cleavage. **E)** $CaCl_2$ activated calpain I induced TRPC1 cleaved fraction at ≈60kDa in HS-5 cells. Pre-treating

cells with calpeptin (80 μM) for 1 hour abrogated TRPC1 cleavage, while no cleavage was seen with MTI-101 treatment. F) Western blot analysis for TRPC1 expression levels in MM.1s cells. G) Effect of knocking down TRPC1 in MM.1s cells on cell death mediated by MTI-101 (30 μM) at 1 hour ($p<0.05$ One-way ANOVA). H) Single cell tracing for $Ca2+$ influx in Fluo-4-AM loaded MM.1s with siTRPC1 and siControl. Cells were treated with MTI-101 (30 μM) with or without one-hour pre-treatement with calpeptin (80 μM). Cells imaged every 30 seconds for one-hour. Mean maximum peak of $Ca2+$ influx in 50 cells in MM.1s cell line. Error bars represent SEM ($p < 0.05$ One-way ANOVA, inter-group comparison was done by Tukey's multiple comparisons test $p < 0.0001$).
